# Epidemiological characterization of *Plasmodium falciparum *in the Republic of Cabo Verde: implications for potential large-scale re-emergence of malaria

**DOI:** 10.1186/1475-2875-5-32

**Published:** 2006-04-21

**Authors:** Joana Alves, Ana Luísa Roque, Pedro Cravo, Tomás Valdez, Tomas Jelinek, Virgílio E do Rosário, Ana Paula Arez

**Affiliations:** 1 Direcção Geral de Saúde, Ministério da Saúde, Palácio do Governo, CP 47, Cabo Verde; 2 Centro de Malária e outras Doenças Tropicais, Instituto de Higiene e Medicina Tropical, Universidade Nova de Lisboa, Rua da Junqueira, 96, 1349-008 Lisboa, Portugal; 3 UEI Biologia Molecular, Instituto de Higiene e Medicina Tropical, Universidade Nova de Lisboa, Rua da Junqueira, 96, 1349-008 Lisboa, Portugal; 4 Berlin Institute of Tropical Medicine, Spandauer Damm 130, 14050 Berlin, Germany

## Abstract

**Background:**

Malaria has come near eradication at archipelago of Cabo Verde in 1970. Infections are now only observed in Santiago, where outbreaks occur. In these islands, malaria is considered by the international community as being of limited risk and, therefore, no prophylaxis is recommended. Since the understanding of factors that determine malaria outbreaks are crucial for controlling the disease, the present study aimed to investigate if the malaria infections observed in Santiago Island are maintained in isolated foci and in asymptomatic individuals.

**Methods:**

The occurrence of asymptomatic carriers in villages with history of malaria as well as the level of exposure of these populations were investigated using PCR and serological analyses.

**Results:**

Results indicate that malaria is maintained as asymptomatic and sub-patent infections and that the majority of the circulating parasite populations harbour chloroquine-resistant mutations.

**Conclusion:**

These observations highlight the alarming prospect of malaria to become a serious public health problem and underscore the need for a tighter surveillance.

## Background

Malaria was almost eradicated from the archipelago of Cabo Verde following a sustained control program between 1940 and 1970. Infections are now only observed in Santiago Island and according to WHO [1], there is only a limited malaria risk between September and November and there is no recommendation for prophylaxis. The population is considered to be susceptible to malaria and irregular outbreaks occur, which are in some instances thought to be related to the influx of immigrants from the African continent. An outbreak in 1995/96 in Santa Catarina district was followed-up and characterized through parasitological and molecular analysis during one year [2]. It was confined to an isolated village resulting in the infection of at least 40% of the villagers with a genetically homogeneous *Plasmodium falciparum *chloroquine-resistant (CQR) parasite. One year after the outbreak, 10% of the inhabitants still harboured parasites of the same genotype. In that study, the potential of chronic malaria carriers to transmit malaria long after the initial infection was demonstrated since gametocytes were still found in the blood of some of the villagers one year later. It was likely that the initial outbreak resulted from the simultaneous occurrence of two conditions that favoured transmission: (i) the presence of infectious gametocytes in at least one individual and (ii) the existence of climatic conditions that allowed the mosquito population to thrive and spread the parasites to the remainder population. This would have implied the existence of a single primary case, a highly susceptible population and a high basic reproductive rate of the parasite, which collectively determine a high transmission potential.

The understanding of malaria epidemiology and factors that determine such outbreaks are crucial for malaria control, especially in areas as Cabo Verde where conditions allow the maintenance of vector populations, still present in some other islands of the archipelago [3], in close proximity to susceptible host populations. The present study aimed to investigate the possibility that the malaria infections observed in Santiago Island are maintained by asymptomatic individuals in isolated and residual foci. The potential occurrence of asymptomatic carriers in villages with history of malaria as well as the level of exposure of these populations was studied through PCR and serological analyses.

## Methods

### Study area

The Republic of Cabo Verde is an archipelago comprised of 10 islands located in the Atlantic Ocean, 500 km West of Senegal. Santiago Island is the largest island, where approximately half of the population resides and where Praia, the capital of the Republic, is situated.

Climate is characterized by a dry season (December-June) and a wet season (July-November) with short and irregular rainfalls.

The malaria vector is thought to be *Anopheles arabiensis*, the only member of the *gambiae *complex recorded on the archipelago since 1982 [2,4,5].

**Figure 1:**
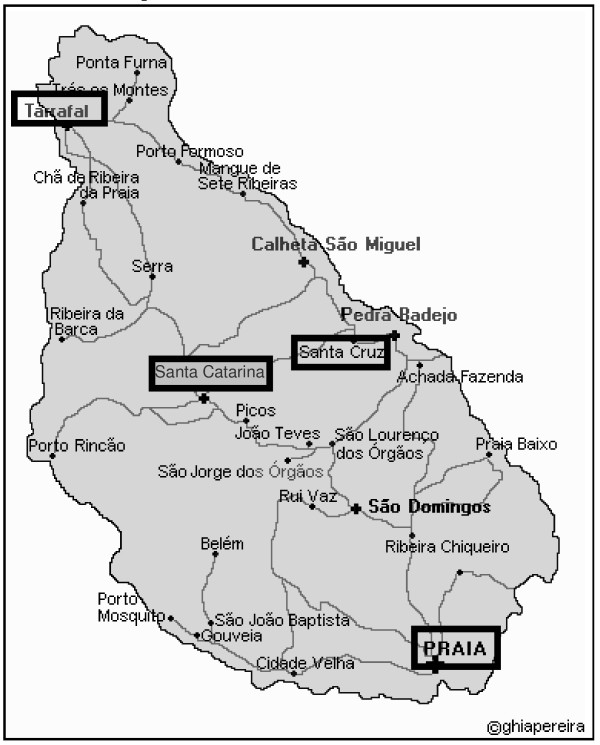
Location of studied districts in Santiago Island, Cabo Verde (adapted from ).

The study was conducted in localities with malaria history in the last six years and localities with limited health care facilities: District of Praia (Palmarejinho, Meio da Achada, Tira Chapéu, Bela Vista, Várzea), District of Santa Cruz (Chã da Silva, Órgãos, Santa Cruz), District of Santa Catarina (Chã de Tanque, Mato Sanches, Engenho, Assomada, Ribeira da Barca) and District of Tarrafal (Chã Bom, Porto Formoso, Vila, Calheta) (Figure 1).

### Sampling

Blood sample collections were carried out from a total of 730 individuals from all ages. A total of 131 individuals, who reported to the local health care facilities with symptoms of headache and fever, were sampled by passive case detection (PCD): 65 between 1998 and 2000 and 66 in 2003). A total of 599 individuals (338 between 1998 and 2000 and 261 in 2003) were sampled by active case detection (ACD) from households, according to the following criterion: in each locality, households with confirmed recent malaria history were first selected and sampled and used to establish a transect parallel to the widest part of the village. The remaining households in the transect were indexed to this one and sampled following a frequency which depended on village size (every 5^th^, 8^th ^or 10^th ^house indexed to the originally selected, in villages clustered as small, medium or large respectively). Sample collections were made from all inhabitants within each household. A questionnaire was filled in for all individuals, including information on symptoms, malaria history, travel inside and outside the island, type of housing, presence of animals and/or mosquito breeding sites in/close to the house.

Between 1998 and 2000, finger-prick collected blood was spotted in Whatman^® ^#4 for DNA preparation and Giemsa stained blood smears were done for microscopical examination. In 2003, blood samples were obtained by venopuncture and used as above and for serological assays.

The investigation was approved by the Ministry of Public Health of Cabo Verde and by the Ethical Committee at Institute of Hygiene and Tropical Medicine of Lisbon. Each person (or parent) was informed of the nature and aims of the study and told that participation was voluntary.

### *Plasmodium falciparum *detection and genotyping

Parasite densities were determined in Giemsa-stained blood smears and recorded as the number of parasites/μl of blood, assuming an average leukocyte count of 8,000/μl (all smears were examined against 500 leucocytes prior to be declared negative). Parasite DNA was obtained from all samples by boiling in Chelex-100 [6] followed by an ethanol precipitation. *P. falciparum *detection was performed by nested-PCR amplification of the small subunit ribosomal RNA genes [7].

*P. falciparum *characterization was carried out in all positive samples for this species by nested-PCR amplification of the block 2 of merozoite surface protein 1 gene (*msp1*), the repeat region of merozoite surface protein 2 gene (*msp2*) and the region RII of glutamate rich protein gene (*glurp*) as previously described [8]. All PCR products were analyzed in the same agarose gel (MetaPhor, FMC Bioproducts) and a final reading of band sizes was done for each allelic family in all gels in order to assure a coherent classification of alleles.

Genotyping of the chloroquine resistance marker *pfcrtK76T *was carried out by PCR-RFLP. A fragment of the *pfcrt *gene containing codon 76 was first obtained by PCR amplification using a novel "Nested" approach, conceived to overcome spurious PCR bands on agarose gels, thus increasing the specificity and sensitivity of amplification. Consequently, several primer pairs were designed and tested, of which only those consistently producing single PCR products were chosen for downstream analyses. All primer sequences and respective PCR amplification conditions are presented in Table 1.

**Table 1:** PCR amplification of DNA fragments harbouring codon 76 of the *pfcrt *gene

**Primer pair (5'-3')**	**Expected fragment size**	**Cycling conditions**	**Reagent concentrations**
**Outer** Sense (O1F) CCTTGTCGACCTTAACAGATG Antisense (N1R) GACTGAACAGGCATCTAACATG	528 bps	Hot Start: 94°C, 3' 40 cycles at: denaturing: 94°C, 45" annealing: 55°C, 45" extension: 72°C, 45" final extension: 72°C, 3'	1 μM/primer, 1 × PCR buffer (Promega ™), 2.5 mM MgCl_2_, 0.2 mM dNTP's, 0.025 U/μl of Promega ™ *Taq *DNA polymerase
	
**Nested** Sense (N2F) ATGGCTCACGTTTAGGTGG Antisense (N2R) CTTTTGAATTTCCCTTTTTATTTCC	271 bps	Hot Start: 94°C, 3' 35 cycles at: denaturing: 94°C, 45" annealing: 53°C, 45" extension: 72°C, 45" final extension: 72°C, 3'	1 μM/primer, 1 × PCR buffer (Promega ™), 2.5 mM MgCl_2_, 0.2 mM dNTP's, 0.025 U/μl of Promega ™ *Taq *DNA polymerase

Following nested amplification of the *pfcrt *fragments harbouring codon 76, the presence of triplets AAA, encoding lysine (K) or ACA, encoding threonine (T), was detected by incubation of the corresponding PCR fragments with endonuclease *ApoI *(r/aatty). The amplified segment contained both a monomorphic and a polymorphic restriction site; *ApoI *thus excised products containing AAA, generating 3 fragments of 137, 124 and 10 bps while PCR products harbouring the alternative allele ACA were cut only at the monomorphic site, resulting in two fragments of 261 and 10 bps.

Endonuclease *ApoI *was obtained from New England BioLabs^® ^and incubations were setup following the manufacturer's instructions. Appropriate control DNA of samples with known *pfcrt *sequences was used in parallel with field-collected parasite isolates in every PCR-RFLP protocol; these were *P. falciparum *3D7 (genotype *pfcrt *76K) and Dd2 (genotype *pfcrt *76T). The products resulting from restriction digests were resolved in 2% agarose gels, made up in 1 × TBE buffer. All gels were stained with ethidium bromide and visualized under UV (ultraviolet) transillumination.

The characterization of all above mentioned markers was simultaneously attempted for samples originated from Praia, Santa Catarina, Santa Cruz and Tarrafal, collected in three different periods: 1995/96, during the malaria outbreak in Santa Catarina [2] and Tarrafal (unpublished data), 1998/2000 (Praia, Santa Catarina, Santa Cruz) and in 2003 (Praia, Santa Catarina, Santa Cruz and Tarrafal).

### Serological analysis

The humoral response against the CSP antigen of *P. falciparum *was evaluated in 296 serum samples collected in 2003, with a NANP19 antibody-test [9]. Each sample was tested between two and four times. A sample was considered to be positive when the average of the absorbance of replicates was higher than three.

## Results

### Infection detection and parasite characterization

Prevalence of infection was different depending on the type of sampling and technique used for parasite detection. In the period 1998/2000, a much higher prevalence of infection was determined by Passive Case Detection; however, both Optical Microscopy (OM) and PCR presented the same trends 59% by PCD (n = 39; 26 individuals without optical microscopy readings were excluded from the analysis) both by OM and PCR and 0 and 1% by ACD (n = 338), by OM and PCR respectively). In 2003, a similar prevalence was determined, independently of case detection method; PCR detected a few infections not detected through OM [PCD (n = 66): 3% by OM and 5% by PCR; ACD (n = 261): 0% by OM and 4% by PCR].

Apparently infections distributed similarly among the studied districts (Table 2) and prevalence was similar in all age groups (slightly higher in the 5–14 years-old age group in 1998/2000; Table 3).

**Table 2:** Prevalence of infection (%) as detected by PCR, according the district and sampling period.

**Sampling period**		**1998/2000**	**2003**
**Overall**		8 (n = 403)	4 (n = 327)
**District**	**Praia**	2 (n = 236)*	4 (n = 132)
	**Santa Cruz**	11 (n = 18)^§^	0 (n = 67)
	**Santa Catarina**	17 (n = 149)	8 (n = 80)
	**Tarrafal**	ND	6 (n = 48)

* All samples collected by Active Case Detection; ^§ ^All samples collected by Passive Case Detection

**Table 3:** Prevalence of infection (%) as detected by PCR, according the age group, case detection method and sampling period.

**Sampling period**		**1998/2000**			**2003**		
**Age group**		**0–4**	**5–14**	**>14**	**0–4**	**5–14**	**>14**

**Case detection method**	**PCD**	14 (n = 7)	55 (n = 29)	31 (n = 29)	0 (n = 25)	0(n = 10)	10 (n = 31)
	**ACD**	2 (n = 63)	3 (n = 166)	1 (n = 29)	0 (n = 28)	4 (n = 93)	5 (n = 137)
	**Total**	3 (n = 70)	14 (n = 138)	5 (n = 195)	0 (n = 53)	4 (n = 103)	6 (n = 168)

PCD – Passive Case Detection; ACD – Active Case Detection.

Parasite densities ranged from 500 to 500,000 parasites/μl with a geometric mean of 23,907.77 and a median of 23,375.00 parasites/μl. All OM positive cases were symptomatic, exhibiting only mild clinical symptoms.

Genetic characterization of genes *msp1*, *msp2 *and *glurp *was achieved in 36 samples (out of 45 samples infected with *P. falciparum*). Regarding Multiplicity of Infection (MOI-the number of distinct genotypes detected in an isolate), single infections predominated (81%) with 22 cases from PCD, (17 in 1998/00 and five in 2003) and seven cases from ACD (five in1998/00 and two in 2003). Dual infections were detected in six cases (five from PCD in 1998/00 and one from ACD in 2003); only one case with three genotypes was found in 2003, in Praia by ACD.

In 1998/2000, dual infections were all observed in the district of Santa Catarina by PCD and in 2003, the 2 multiple infections were found in the districts of Praia (MOI = 3) and Tarrafal (MOI = 2).

Regarding the genetic diversity of these three genes, the IC allelic family was the most prevalent for the *msp2 *gene (with 7 different alleles) and the K1 for the *msp1 *gene with only one allele; three different alleles were found for *glurp*.

In 1998/2000, in Praia, the same parasite genotype was found in the four *falciparum*-infected isolates; these corresponding to four individuals, found by ACD, living in the same neighbourhood, of which three living in the same house. These genotypes are different from those harboured by the three isolates collected in 2003 in which a successful typing was achieved (two of them, found by PCD, lived in the same house and exhibited the same parasite genotype). However, the only allele *msp1*-MAD20(200) was found in this district and at both dates of collection.

In 1998/2000, similar parasite genotypes were detected in Santa Catarina (25 isolates) and Santa Cruz (two isolates). Unfortunately, in 2003, parasite characterization could not be achieved in the isolates of Santa Catarina and no infections were detected in Santa Cruz. Parasite genotypes were different from those collected during the outbreak in Santa Catarina in 1995.

The isolates collected in Tarrafal, in 1995/96 (from two individuals living in the same house infected with the same parasite genotype) and 2003 (from three individuals), had different genotypes and were different from everywhere else.

**Figure 2:**
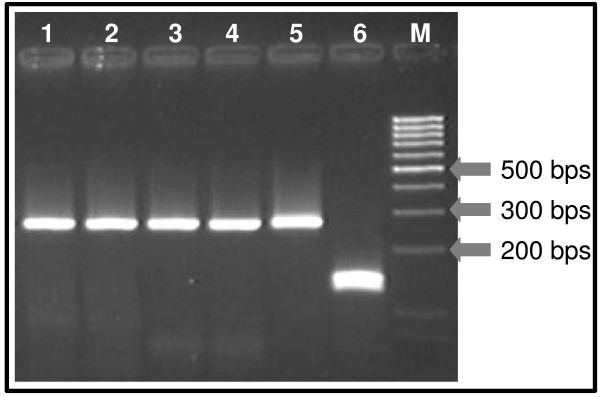
**Agarose gel depicting results of *pfcrt*76 genotyping**. (Lanes 1 to 4: *P. falciparum *field-collected isolates harboring *pfcrt *76T mutant alleles; lane 5 *P. falciparum *K1 control DNA; lane 6: *P. falciparum *3D7 control DNA; lane M: DNA ladder "low-range", Fermentas™).

For *pfcrt *genotyping, after optimization of PCR-RFLP reactions, the assay proved to be specific in discriminating *pfcrt *alleles encoding 76K or 76T, as determined by the correct genotyping of DNA originated from *P. falciparum *3D7 and K1 respectively. After restriction digestion with *ApoI*, the presence of the K-encoding triplet AAA, generated 3 products of 137, 124 and 10 bps; the 10 bp fragment is undetected on agarose gels, while the 137 and 124 bp products migrated as a single band. PCR products harbouring the allele ACA (coding for T) resulted in two fragments of 261 and 10 bps. Typical results can be visualized in Figure 2.

The technique was applied to genotype field isolates. Successful typing was achieved for 41 out of 51 samples included in the study, previously determined to be malaria-positive. PCR amplifications were unsuccessful in 10 samples, all belonging to the same sampling cluster, possibly due to DNA degradation after sample collection or DNA extraction.

The major findings revealed that both wild-type 76K and mutant alleles, 76T, were present in the circulating parasite population, with the latter being found at significantly higher prevalence. The overall results are summarized in Table 4.

**Table 4:** *Pfcrt*76 genotypes according to geographical distribution and collection period.

	**Geographical origin**							
**Collection period**	**Praia**		**Santa Cruz**		**Santa Catarina**		**Tarrafal**	
	76K	76T	76K	76T	76K	76T	76K	76T

**1995/96 **(n = 6)	ND		ND		0	4	2	0
**1998–2000 **(n = 29)	0	4	0	2	0	23	ND	
**2003 **(n = 26)	3	0	NI		UT		0	3
Successfully typed/malaria-positive	7/7		2/2		27/37		5/5	

ND – not done; NI – non-infected; UT – unsuccessful typing.

### Serological analysis

Seropositivity for anti-CSP antibody was detected in 7% of the PCD (n = 61 samples) and in 9% of the ACD (n = 235 samples) isolates. Regardless of case detection method, serology detected anti-CSP antibodies in 21% of the current infections (as detected by PCR; n = 14 samples) and in 6% of the non-infected individuals who reported a history of malaria in the last 6 months (n = 126 samples).

Seroprevalence was stratified by age, but no significant difference was found between age groups (p > 1.10) or between the studied districts (p > 0.08).

## Discussion

Cabo Verde, especially the island of Santiago, is presently an area of epidemic malaria, where irregular outbreaks occur. In a previous study [2], one of these outbreaks was studied and the following features were identified: a) most of the infected persons only developed mild clinical symptoms, in all age groups, in spite of the presumably high susceptibility of the population; b) for the first time, a genetically homogeneous parasite population was found to be in circulation around the island; c) infections persisted for at least one year in 10% of the population and gametocytes were observed throughout the study period (potential of chronic malaria carriers to transmit malaria long after the initial infection). Data regarding the bio-ecology of the mosquito vector populations is lacking and it was only possible to obtain an indirect assessment of mosquito-mediated transmission [10].

Since the understanding of malaria epidemiology and factors that determine outbreaks are crucial to design the most appropriate malaria control strategy, the present study aimed to investigate whether the malaria infections observed in Santiago Island are being maintained in isolated and residual foci in asymptomatic carriers.

In the period 1998/2000, a much higher prevalence of infection was determined by PCD than by ACD; however, both optical microscopy and PCR presented the same trends. In that period, the diversity of sample sources (different case detection methods in different localities) may have acted as confounders. Thus, in 2003 a more systematic sampling procedure was carried out. This revealed a similar prevalence, independently of case detection method, but PCR allowed the detection of a few infections undetected by microscopy.

As in the previous study [2], no complicated malaria cases were found in spite of high parasitaemia. Usually it is considered that a clinical malaria attack occurs at cut-off levels of parasitaemia in the range of 1,000–10,000 parasites/μl [11,12]. In this study, from the 26 cases with patent parasitaemia, 20 were above this limit, although individuals presented no more than mild symptoms such as fever, headache, nausea and general malaise. Thus, it appears that individuals did not develop severe symptoms of malaria despite exposure to the blood stages of the parasite and many even seem to have a subclinical course. These observations are consistent with similar studies carried out in other areas of unstable and low-level transmission malaria [13,14]. Despite the low prevalence of malaria, the establishment of clinical symptoms is attenuated, probably because of the acquisition of premunition. Other possible explanations for the low morbidity associated with malaria infections in this island may be related with genetic factors of both parasites and host, which may control the outcome of malaria infections.

No evidence is available regarding the contribution of parasite genetic factors or different ability to induce clinical disease (virulence) [12] to the low morbidity observed in the island. In the previously studied and much localized outbreak Sta Catarina district, we could only suggest that the genetically homogeneous circulating parasite population described could have been a weakly virulent parasite. However, in this study where different localities were studied and heterogeneity in the diversity of *P. falciparum *was detected this assumption would be very speculative.

Also, considerable differences in clinical consequences of infection with *P. falciparum *as consequence of host factors have already been demonstrated [15,16]. Research on this subject is presently going-on since there is no data available for the Cabo Verde archipelago.

In areas such as Cabo Verde, serological parameters offer a theoretical advantage over parasite prevalence as a measure of endemicity, in that antibodies can persist for months or years after infection, thereby smoothing out the effects of seasonal or unstable malaria transmission. Serology offers theoretical advantages over entomological and parasitological measures of malaria transmission in that a single measurement reflects exposure over an extended period, thus overcoming the sampling bias associated with seasonal variation in transmission levels and year-to-year variations in the timing and extent of peak transmission [17]. Seroepidemiology may also be used to identify local *foci *in a given area [18,19] and seroprevalence may be a very useful tool to assess the results of a malaria control programme. The slow reversion rate of seropositivity would be of little impact in adults or older children but significant changes in seroconversion rates in infants and younger children born after the implementation of the programme should occur, implying that age stratification of the seroprevalence data is important.

In the present study, seroprevalence to circumsporozoite protein was low. Antibody levels to whole parasites or to individual antigens may persist even in the absence of periodic or subpatent infection. Further, if infections persist as it seems to occur, based on parasite detection data, frequent or persistent subpatent malaria infection is sufficient to maintain seropositivity, in the absence of effective antimalarial treatment [20]. In this study, anti-CSP antibody detection was of low value in face of the high number of seronegatives among the infected which may reflect the low sensitivity of the assay [9]. However, acutely infected patients may become positive in the weeks after the onset of symptoms and a report on history of malaria may not be reliable. Probably, other serological markers should be added to estimate exposure (e.g. testing for anti-merozoite-antibodies). When these markers are inappropriate they might underestimate it because of lack of sensitivity, especially in infants [21] and in fact, no difference was found between infants, children and adults.

In Cabo Verde, chloroquine has been the first line treatment for *falciparum *parasites for several decades. Although no *in vivo *data on the outcome of chloroquine treatment in these islands is available, an early *in vitro *study carried out in 1995/96, revealed IC50 values suggestive of chloroquine resistance [2]. Although in the present survey, the parasite population was not phenotyped for its response to chloroquine, the chloroquine-resistance associated marker *pfcrt*76T was found at much higher frequencies than its wild-type counterpart. Collectively, the aforementioned data is indicative of a CQR phenotype among the majority of the circulating parasite population. Drug misuse by islanders has possibly been the major selective force driving the maintenance of these parasites, although all data available is insufficient to infer whether chloroquine resistance was selected *de novo *or externally introduced from continental Africa.

The relationship between levels of transmission and endemicity and the dynamics of drug resistance in malaria has been subject of debate. In Cabo Verde malaria transmission and endemicity levels are low, which according to recent views may actually favour both the emergence and maintenance of drug resistance in malaria parasites [22].

## Conclusion

As it is characteristic of an unstable and low-transmission malaria area, in Santiago island, Cabo Verde, malaria infection is found in all age groups. Apparently, *P. falciparum *survives the long, dry and transmission-free periods as asymptomatic sub-patent infections, and resurgences occur after abundant rains and a consequent increase of the vector population.

The maintenance of the malaria infections observed in Santiago Island in isolated *foci *was not clearly demonstrated since both infection and seroprevalence distributed similarly among the studied districts and analysis of parasite genotypes were not conclusive. Further studies in other localities of the island are still required.

Until the present, malaria has been considered by the international community as being of limited risk and no prophylaxis is usually recommended. However, our studies indicate that malaria infections are maintained along the island and regardless of the resistance dynamics, the observation that CRQ parasites are highly frequent among the population of Santiago is highly relevant in terms of malaria control policies. Altogether, these results, namely the 1) sub-patent carriage of infections, 2) the potential to transmit long after the initial infection, as observed in the previous study [2] and 3) the co-existence of drug-resistant parasites are thus factors that may favor an outbreak of malaria of potentially difficult control and disastrous consequences if any environmental change implies an increase in the transmission level. According the reports of the National Programme against Malaria ("Programa Nacional de Luta contra o Paludismo" PNLP) [23], the number of reported malaria cases in the last years has increased and environmental changes have been observed. Results gathered in the present work and these PNLP's observations stress the need of a continuous and strong surveillance with establishment of sentinel-units in vulnerable localities and periodic serological inquiries complemented with parasite detection with sensitive techniques.

## Authors' contributions

JA conceived the study, carried out the sample collections, the laboratory analysis and the analysis and interpretation of data. ALR carried out the genotyping of the chloroquine resistance marker. PC designed the *pfcrtK76T *genotyping method and helped to draft the manuscript. TV participated in the data collection in the field. TJ performed the immunoassays. VER participated in the analysis and interpretation of data. APA participated in the study design and coordination, in the analysis and interpretation of data and drafted the paper. All authors read and approved the final manuscript.
